# A Graphene/Polycrystalline Silicon Photodiode and Its Integration in a Photodiode–Oxide–Semiconductor Field Effect Transistor

**DOI:** 10.3390/mi11060596

**Published:** 2020-06-17

**Authors:** Yu-Yang Tsai, Chun-Yu Kuo, Bo-Chang Li, Po-Wen Chiu, Klaus Y. J. Hsu

**Affiliations:** Institute of Electronics Engineering, National Tsing Hua University, Hsinchu 30013, Taiwan; st95410@gmail.com (Y.-Y.T.); kuochenyou@gmail.com (C.-Y.K.); pet791228@gmail.com (B.-C.L.); pwchiu@ee.nthu.edu.tw (P.-W.C.)

**Keywords:** graphene, polycrystalline silicon, photodiode, phototransistor, pixel, high dynamic range (HDR) image

## Abstract

In recent years, the characteristics of the graphene/crystalline silicon junction have been frequently discussed in the literature, but study of the graphene/polycrystalline silicon junction and its potential applications is hardly found. The present work reports the observation of the electrical and optoelectronic characteristics of a graphene/polycrystalline silicon junction and explores one possible usage of the junction. The current–voltage curve of the junction was measured to show the typical exponential behavior that can be seen in a forward biased diode, and the photovoltage of the junction showed a logarithmic dependence on light intensity. A new phototransistor named the “photodiode–oxide–semiconductor field effect transistor (PDOSFET)” was further proposed and verified in this work. In the PDOSFET, a graphene/polycrystalline silicon photodiode was directly merged on top of the gate oxide of a conventional metal–oxide–semiconductor field effect transistor (MOSFET). The magnitude of the channel current of this phototransistor showed a logarithmic dependence on the illumination level. It is shown in this work that the PDOSFET facilitates a better pixel design in a complementary metal–oxide–semiconductor (CMOS) image sensor, especially beneficial for high dynamic range (HDR) image detection.

## 1. Introduction

The unique structural, electronic, and optical properties of graphene have made this two-dimensional material an attractive subject of research in recent years. While the high carrier mobility in graphene opened the door to its applications in high-speed electronics [1,2,3,4], the application of graphene in optoelectronics has also become an interesting and important topic. The linear dispersion of the Dirac electrons in graphene can be utilized for broadband optical detection, and the intrinsic speed of the photodetectors can be very high. Unfortunately, using single-layer graphene for vertical optical absorption led to very low photocurrent responsivities [5,6,7,8], normally below or about 10 mA/W, because of the high transparency of graphene over a wide band of light wavelengths [9,10]. Many interesting strategies have been proposed to enhance the optical absorption by the graphene in graphene-based photodetectors. They require specially patterned structures such as waveguides [11], resonant cavities [12], plasmonic nanostructures [13], graphene nanodisks [14], or graphene quantum dots [15,16], and the resultant photocurrent responsivities have been successfully increased. For more responsive optical detection in simple planar, single-layer graphene-based photodetectors, a natural way is to construct a hybrid device in which the role of optical absorber is left to other materials in the device and the graphene is used as an efficient conductive material for carrier collection. Graphene has been adopted to form transparent electrodes in various solar cells [17,18,19,20]. It has also been extensively combined with crystalline silicon (c-Si) and germanium to form Schottky photodiodes [21,22,23,24,25,26,27,28,29,30,31,32]. In these cases, the transparency of graphene becomes an advantage. Incident light is not blocked by the graphene, and the spectral photocurrent responsivities of the devices are determined by the other combined materials instead of the graphene. Taking graphene/c-Si junction photodetectors as an example, the c-Si is the optical absorber and photocurrent responsivities in the order of 100 mA/W are typically obtained. The performance is similar to that of typical c-Si P/N junction photodiodes and is good enough for numerous applications.

Interestingly, while there have been a number of studies studying graphene/c-Si junctions and their application to photodetection [21,22,23,24,25,26,27,28,29,30,31], the study of the graphene/polycrystalline silicon (graphene/poly-Si) junction and its application is rare in the literature. Only Lin et al. have investigated the change in the current density-voltage (J-V) characteristic of a four-layer graphene/p-type poly-Si junction after being influenced by some ultraviolet illumination [33]. No study on single-layer graphene/n-type poly-Si junctions has been reported. However, it may be beneficial to pay attention to this kind of junction because it could have application potential on occasions when poly-Si exists. Metal–oxide–semiconductor field effect transistors (MOSFETs) with a poly-Si gate and poly-Si thin film solar cells are two examples of possible applications. In the present work, a junction made of single-layer graphene and n-type poly-Si was fabricated and characterized. Furthermore, a new phototransistor named the PD–oxide–semiconductor field effect transistor (PDOSFET), in which the graphene/poly-Si junction photodiode (PD) is embedded in a conventional metal–oxide–semiconductor field effect transistor (MOSFET), was proposed and experimentally verified. Analysis showed that such phototransistor is particularly useful in constructing the pixels in complementary-metal–oxide–semiconductor (CMOS) image sensors for detecting high-contrast images in which the brightness is of high dynamic range (HDR).

## 2. Fabrication and Characterization of Graphene/Poly-Si Junction

**Figure 1:**
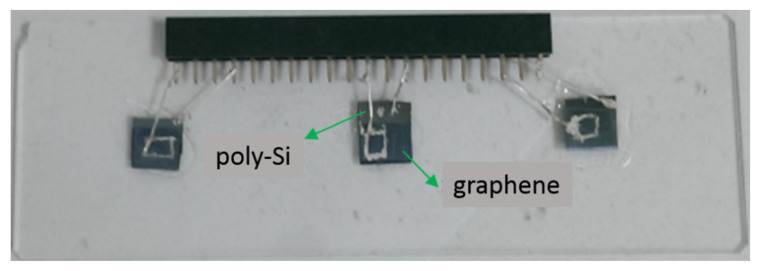
Samples of graphene/poly-Si junction. The graphene film partially covers the poly-Si in each sample.

Before a graphene/poly-Si junction is used for applications, it is essential to have a look at its general electrical and optoelectronic characteristics. For this purpose, samples of graphene/n-type poly-Si junction were fabricated. A 300 nm-thick phosphorus-doped poly-Si film was deposited on an n-type Si wafer by using low pressure chemical vapor deposition (LPCVD) at a temperature of 620 °C. Because the optical absorption coefficient of poly-Si in the visible light band is between 10^4^ and 10^5^ cm^−1^ [34], the thickness (300 nm) of the poly-Si was chosen to ensure that most of the incident photons could be absorbed when the film was illuminated by visible light. The graphene film was first grown on a copper foil by using ambient pressure chemical ovapor deposition (APCVD) at a temperature of 1040 °C and was subsequently transferred onto the poly-Si by using a commonly adopted graphene wet transfer process [35,36]. The poly-Si was partially covered by the transferred graphene film. Afterwards, the wafer was cut into discrete devices, each with an area of about 25 mm^2^. Silver paste was respectively applied to the surface of exposed poly-Si and the surface of graphene to form the two electrical terminals of the graphene/n-type poly-Si junction. The reason for using silver paste as the contact material was that it could be easily applied to quickly finish sample preparation and it had been tested to form ohmic contacts with graphene and with n-type c-Si. It was speculated that the paste might still form ohmic contacts with n-type poly-Si. Figure 1 shows a photo of typical samples placed on glass.

**Figure 2:**
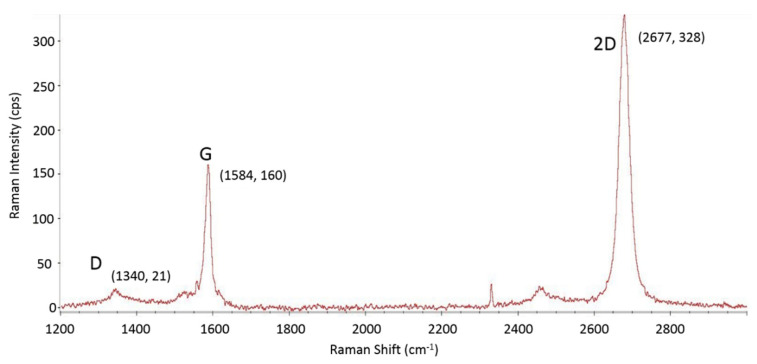
Raman spectrum of the transferred graphene layer. It indicates that the graphene is a single layer with slight defects.

The sheet resistance (*R*_SH_) of the graphene film was measured by using a four-point probe. Values between 250 Ω/square and 300 Ω/square were obtained for all the samples. Figure 2 shows the Raman spectrum of the graphene after the transfer process. From the 2:1 intensity ratio between the 2D and G peaks, it can be deduced that the transferred graphene is of a single layer [35]. The slight D peak indicates that there may be some defects such as wrinkles in the transferred graphene layer.

**Figure 3:**
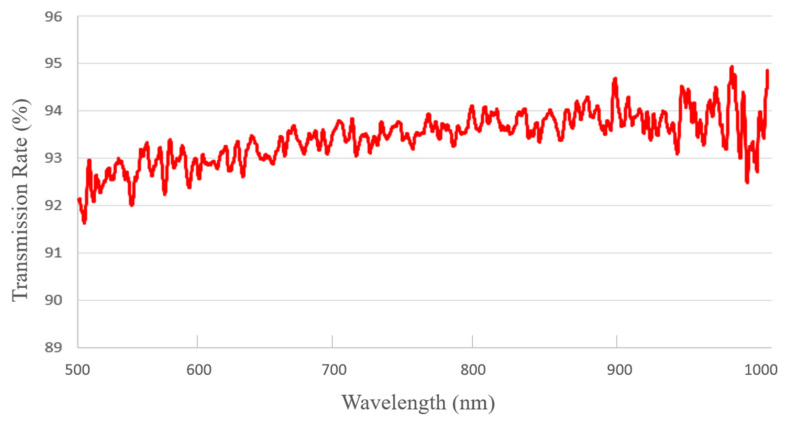
Measured total transmission rate of a typical graphene-on-glass sample. The transmission rate of the glass is about 96%.

Optical measurement was also performed to characterize the optical transmission rates of several transferred graphene samples. Graphene films were placed on glass substrates with a 96% transmission rate. The total transmission rates of the samples were then measured. All the resultant transmission rates of the graphene films are between 97% and 98% over a wide range of optical wavelengths, which is consistent with the single-layer structure of the graphene films. Figure 3 shows a typical measured spectrum of the graphene-on-glass assembly.

**Figure 4:**
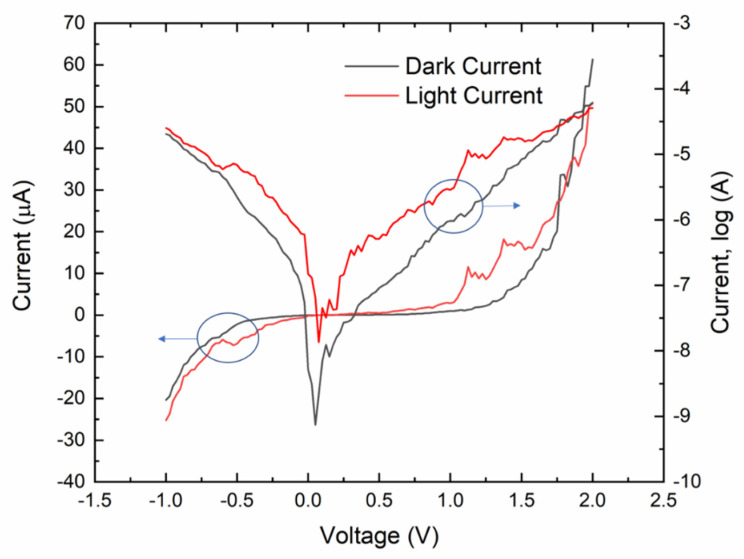
Measured *I*–*V* curves of the graphene/n-type poly-Si junction. The voltage is applied to the graphene terminal and the poly-Si terminal is grounded.

To see the graphene/n-type poly-Si junction behave like an ohmic junction or a Schottky junction, the dark *I*–*V* curve of the fabricated graphene/n-type poly-Si junction was measured by using the Agilent 4156a Semiconductor Parameter Analyzer (Agilent, Santa Clara, CA, USA) at room temperature with the terminal of the poly-Si set at ground potential. Figure 4 shows a typical result along with the *I*–*V* curve under the illumination of a halogen lamp. The optical power of the lamp was unknown. It can be seen from Figure 4 that the measured *I*–*V* curves do not show the simple exponential behavior of a single rectifying junction. Instead, they show the typical behavior of a metal–semiconductor–metal (MSM) structure with two asymmetric rectifying junctions [37,38]. The exponential behavior of the curves under negative bias indicates that in addition to the single-layer graphene/n-type poly-Si junction, another rectifying junction with a smaller built-in potential and in the opposite direction exists in the current path. In this case, it should be due to the silver paste/poly-Si junction. Unlike our previously tested silver paste/n-type c-Si junction that showed ohmic conduction, this silver paste/poly-Si junction appears to be a Schottky contact, and it forms an MSM structure along with the single-layer graphene/n-type poly-Si junction. The phenomenon that the current under forward bias increased when light was applied to the sample also confirms this point. The reverse biased silver paste/poly-Si junction contributed a photo-generated electron current to the forward biased graphene/n-type poly-Si junction. If the silver paste/poly-Si junction was ohmic, the forward bias current should have decreased when the light was on. Although the silver paste/poly-Si junction complicates the measured *I*–*V* curves and it is no longer straightforward to extract the exact values of junction parameters (e.g., Richardson constant and Schottky barrier), we can still see that the dark current flowing through the single-layer graphene/n-type poly-Si junction increases exponentially with increasing forward bias voltage. The large ideality factor, *n*, of this disturbed forward bias characteristic was found by curve fitting to be about 10. The exponential dark *I*–*V* curve under forward bias implies that we should be able to see a logarithmic dependence on light intensity from the open-circuit photovoltage of the junction, as analyzed below.

The general exponential dark *I*–*V* characteristic of a junction can be expressed as

(1) $$I_{D}=I_{S}\left[\exp\left(\frac{V_{D}}{V_{T}}\right)-1\right]$$

where $I_{D}$ is the junction current, $V_{T}=\frac{nkT}{q}$, *n* is the ideality factor of the junction, *I_s_* is the dark reverse saturation current of the junction, and $V_{D}$ is the voltage across the junction. When this junction is used as a PD, the short-circuit photocurrent (*I_ph_*) of the PD is directly proportional to the incident light intensity. If at *t* = 0, a light starts to illuminate the junction under the open-circuit condition, the photocurrent does not flow out. It charges the junction capacitance (*C_D_*) and internally forward biases the junction at the same time. The dynamics can be described by

(2) $$I_{ph}-I_{D}=C_{D}\frac{dV_{D}}{dt}$$

Solving Equations (1) and (2) by the separation of the variables, with the zero initial condition $V_{D}\left(0\right)=0$, one can derive that the open-circuit junction voltage under illumination, i.e., the photovoltage (*V_ph_*), should follow the next equation:

(3) $$V_{ph}\left(t\right)=V_{T}\ln\left\{\frac{I_{ph}+I_{s}}{I_{ph}\exp[-\frac{\left(I_{ph}+I_{s}\right)t}{V_{T}C_{D}}]+I_{s}}\right\}$$

where *I_ph_* is the photocurrent, and *t* is the exposure time. The logarithmic response of the photovoltage to light intensity (via *I_ph_*) is advantageous because it mimics human eyes’ response to brightness. Utilizing *V_ph_* as the output signal allows one to detect a wide dynamic range of light intensity. Unlike the photocurrent that may easily saturate the subsequent reading circuit at high illuminance, the photovoltage allows the circuit to function properly because the signal is logarithmically compressed at high illuminance but still linear at low illuminance. When the incident light is weak or the exposure time is short, Equation (3) reduces to

(4) $$V_{ph}≅\frac{I_{ph}t}{C_{D}}$$

which indicates that the photovoltage under the weak illumination condition is a linear function of the incident light intensity, just as the photocurrent is. On the other hand, for strong illumination that produces a large photocurrent, Equation (3) becomes

(5) $$V_{ph}=V_{T}\;\ln(\frac{I_{ph}+I_{s}}{I_{s}})≅V_{T}\;\ln(\frac{I_{ph}}{I_{s}})$$

which shows that the photovoltage is a compressed output signal of the strong incident light intensity. Note that both Equations (4) and (5) are independent of the area of PD since *I_ph_*, *I_s_*, and *C_D_* are all proportional to the junction area, which means that it is not necessary to enlarge the device size of the PD in order to obtain a significant magnitude of photovoltage.

**Figure 5:**
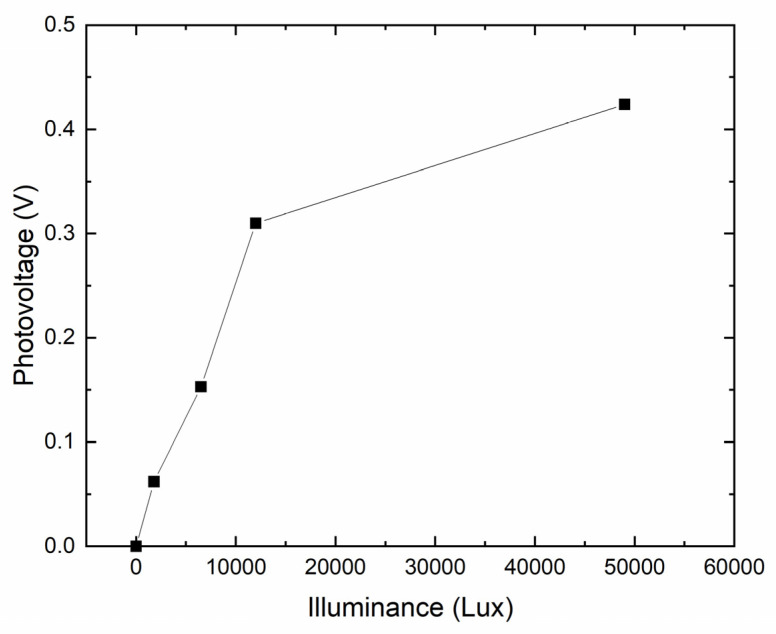
Measured photovoltage of the graphene/n-type poly-Si junction photodiode (PD) under the illumination of a halogen lamp.

Since the measured dark *I*–*V* curve of the fabricated graphene/n-type poly-Si junction sample at room temperature shows an exponential characteristic under forward bias, it can be expected that if this junction is used as a PD, the photovoltage of the PD should exhibit a logarithmic dependence on the incident light intensity. Even though there exists an additional silver paste/poly-Si junction at the other side of the poly-Si, the photo-generated holes in the poly-Si can still flow to the graphene and build up the photovoltage. Figure 5 shows the measured open-circuit photovoltage of a graphene/n-type poly-Si junction sample under the illumination of a halogen lamp. When the illuminance is 49,000 lux, the photovoltage reaches 0.424 V. As expected, the photovoltage increases logarithmically with the illuminance. At higher illuminance, the increase in photovoltage is gradually compressed.

## 3. Photodiode–Oxide–Semiconductor Field Effect Transistor

**Figure 6:**
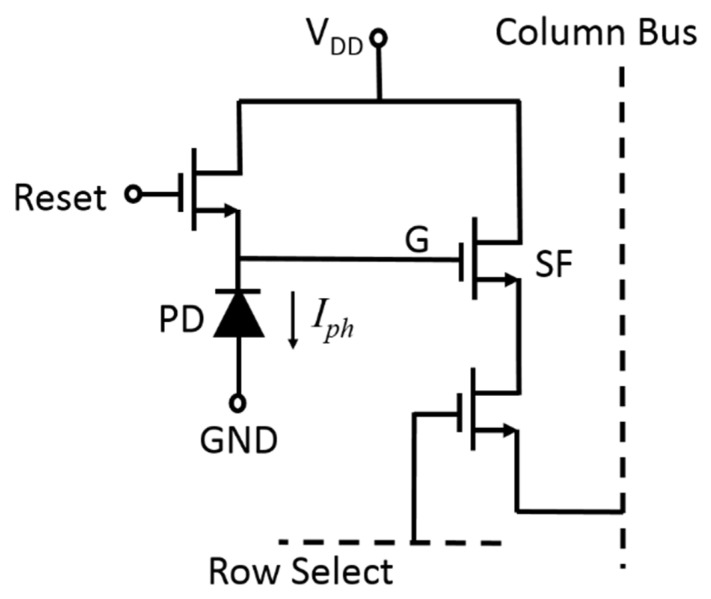
Conventional 3T pixel structure in a complementary-metal–oxide–semiconductor (CMOS) image sensor.

The logarithmic behavior of the photovoltage of a PD makes this variable particularly useful for being adopted in CMOS image sensors as a signal to detect high dynamic range (HDR) images. Furthermore, the graphene/n-type poly-Si junction PD can bring even more benefit to this application because it can be implemented right on top of the gate oxide of a MOSFET in the pixel of a CMOS image sensor. Figure 6 shows the typical structure of a three-transistor (3T) active pixel commonly used in a CMOS image sensor [39,40,41].

Conventionally, the PD in the pixel is a monolithic P–N junction diode located beside the transistors. To operate the pixel, the Reset transistor is first turned on to set the voltage at node G (*V_G_*) to a high value (*V*_DD_). Then, the Reset transistor is turned off and *V_G_* is reduced by the discharging mechanism due to the photocurrent *I_ph_* generated from the PD. The amount of the voltage reduction (Δ*V*) at node G is a linear function of *I_ph_* as shown in Equation (6):

(6) $$\Delta V=\frac{I_{ph}\Delta t}{C_{T}}=\frac{I_{ph}\Delta t}{C_{D}+C_{G}+C_{p}}$$

where Δ*t* is a specified discharging period, *C_T_* is the total capacitance at node G, *C_D_* is the capacitance of the PD, *C_G_* is the capacitance looking into the gate of the source follower (SF) transistor, and *C_p_* is the parasitic capacitance due to interconnect. A large fill factor of the PD in the pixel is preferred so that *I_ph_* can be maximized and *C_T_* can be less influenced by *C_G_* and *C_p_*. After the discharging period,

(7) $$V_{G}=V_{\mathrm{DD}}-\Delta V$$

The *V_G_* signal is output through the source follower to the column bus, and Δ*V* is used to represent the average light intensity during the discharging period. This linear operation principle has limitations: Firstly, for high light intensity, *I_ph_* and Δ*V* can easily become too large and *V_G_* may be reduced too much to make the SF transistor functional. This saturation limits the detectable range of input light intensity. Secondly, for high resolution image sensors, there exists a tradeoff between the pixel size and the fill factor of the PD in a pixel. This limits the values of *I_ph_* and *C_D_*, and the influence from *C_G_* and *C_p_* becomes obvious. Modifying the 3T pixel structure can resolve these limitations.

**Figure 7:**
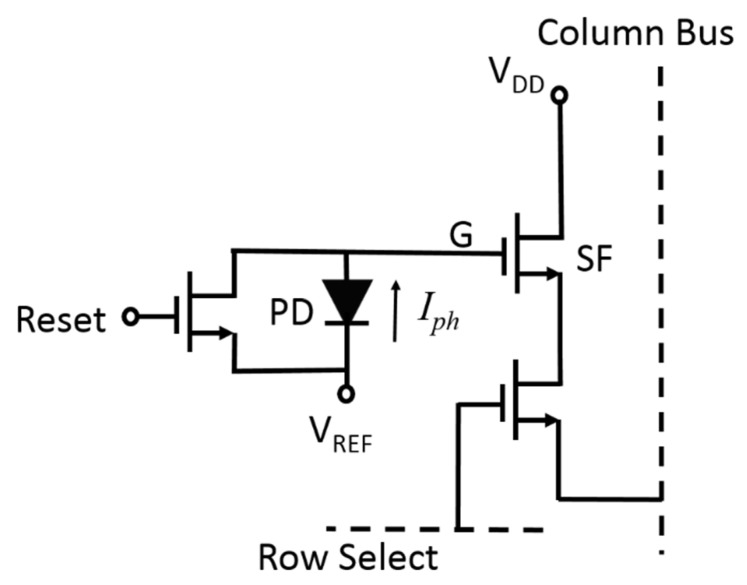
Proposed 3T pixel structure in a CMOS image sensor.

Figure 7 shows the proposed modified 3T pixel structure in which only slight changes from Figure 6 are needed: the location of the Reset switch is changed, the polarity of the PD is reversed, and the ground (GND) potential is replaced by a proper positive bias voltage (*V*_REF_) that makes the SF transistor always functional. In this way, the *V_G_* is first reset to V_REF_ and then becomes the sum of V_REF_ and the photovoltage of the PD after some exposure time *t*. The photovoltage of the PD can be expressed as Equation (3) except that *C_D_* should now be replaced by *C_T_*. Therefore, as explained in Equations (4) and (5), the *V_G_* in the modified pixel structure now behaves linearly at low light intensity but changes logarithmically at high light intensity. With the proper setting of *V*_REF_, the *V_G_* value does not exceed V_DD_ and can always make the SF transistor functional. This natural compression of the signal allows the dynamic range of incident light intensity to be extremely wide, which makes the single-shot detection of high-contrast images possible. There is no need to take multiple shots of an HDR image at different exposure levels and then blend these images together for a composite image. Furthermore, if the original P–N junction PD is replaced with a graphene/n-type poly-Si junction PD that is directly fabricated on top of the gate oxide of the SF transistor, the parasitic capacitance *C_p_* can be eliminated and the tradeoff between pixel size and fill factor can be removed. That is, one can combine the PD and the SF transistor into a new integrated device, namely, the PD–oxide–semiconductor field effect transistor (PDOSFET), to maximize pixel performance. In this way, since the PD is directly stacked on the gate of the SF transistor, metallic interconnect is not needed and *C_p_* is completely eliminated. In addition, because the stacking makes the area of the PD the same as the gate area of the SF transistor, the photovoltage of the PD is independent of the area and there is no more area-competing issue between the PD and the MOSFET. Poly-Si/graphene PD offers unique advantages for this integration. The n-type poly-Si gate is an essential element in most CMOS technologies. All one needs to do is to apply a graphene film to the gate area to form the PD. Additionally, the graphene film has good thermal stability and can withstand the thermal cycles in subsequent fabrication processes. Therefore, one can apply it either before or after the poly-Si deposition process, which allows flexibility in designing the polarity of the PD. If graphene is placed above poly-Si, its transparency allows light to pass through and reach the poly-Si for optical absorption. Metal/poly-Si Schottky PD cannot provide these advantages.

**Figure 8:**
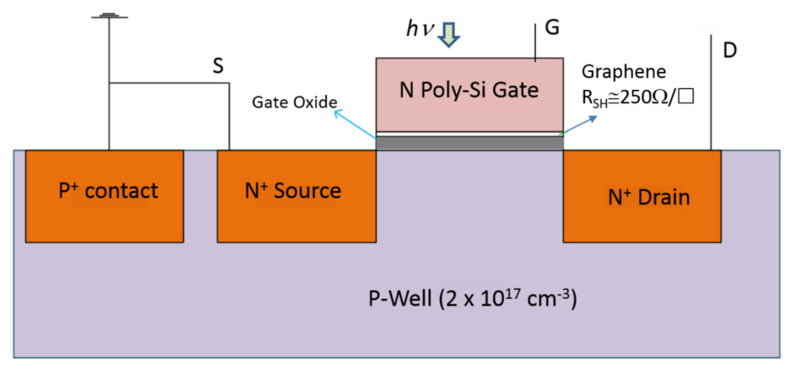
Schematic illustration of a graphene photodiode–oxide–semiconductor field effect transistor (PDOSFET) test device. Poly-Si gate thickness: 300 nm; gate oxide thickness: 20 nm; gate length: 6 μm.

To verify the PDOSFET concept, a test device as schematically illustrated in Figure 8 was fabricated. The channel length was set to be long (6 μm), and the channel width was designed to be 12 μm. The gate oxide was grown by thermal oxidation after the formation of a P-well. It was chosen to be thick (20 nm) to reduce leakage currents in order to assure that the photovoltage developed by the PD would not be degraded. A single-layer graphene was transferred onto the gate oxide before depositing the 300 nm-thick n-type poly-Si gate.

**Figure 9:**
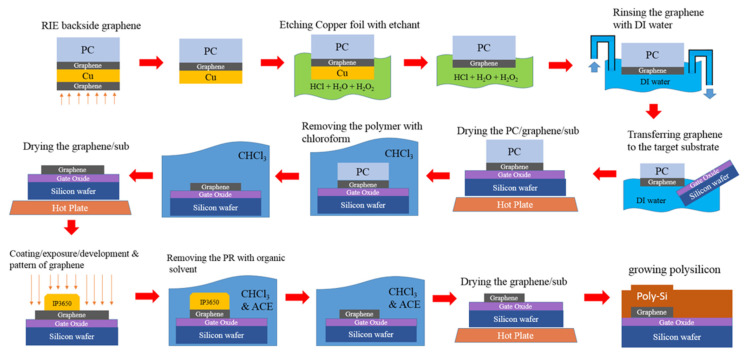
Schematic illustration of the graphene transfer process in fabricating a PDOSFET.

Figure 9 shows the process of transferring the single-layer graphene from copper foil onto the gate oxide. After the graphene was synthesized on the copper foil, it was first supported and protected by a coated poly(bisphenol A carbonate) (PC) layer, and the redundant graphene on the back side of the copper foil was etched away by using reactive ion etching (RIE) with oxygen plasma. Then, the copper foil was removed in an aqueous solution of HCl and H_2_O_2_. After rinsing in de-ionized (DI) water, the graphene was placed onto the Si wafer with gate oxide already grown on it, and the PC layer was subsequently removed with chloroform. The graphene was then patterned by lithography and RIE according to the designed gate pattern, followed by normal poly-Si gate deposition, lithography, and self-aligned CMOS processes in which the source and drain doping was implemented by using arsenic (As) ion implantation with an energy of 30 KeV and a dose of $8\times 10^{13}\;{\mathrm{cm}}^{-2}$. Because front illumination is adopted for the PDOSFET to detect light, the gate contact metal was intentionally designed to cover only a very small portion of the gate area so that light could reach the graphene/n-type poly-Si junction PD. However, both the drain and source regions were fully covered by a metal layer so that their parasitic P–N junction diodes would not intervene during illumination. The ohmic contact to the poly-Si gate was formed by sequentially depositing Ti and Au films. Therefore, the MSM structure observed in the samples shown in Section 2 did not appear in the PDOSFET. The graphene film sustained the subsequent processes. The poly-Si of one sample was removed to expose the underlying graphene for Raman spectroscopic characterization. The same spectrum as that in Figure 2 was observed. The single-layer graphene held its structure well.

**Figure 10:**
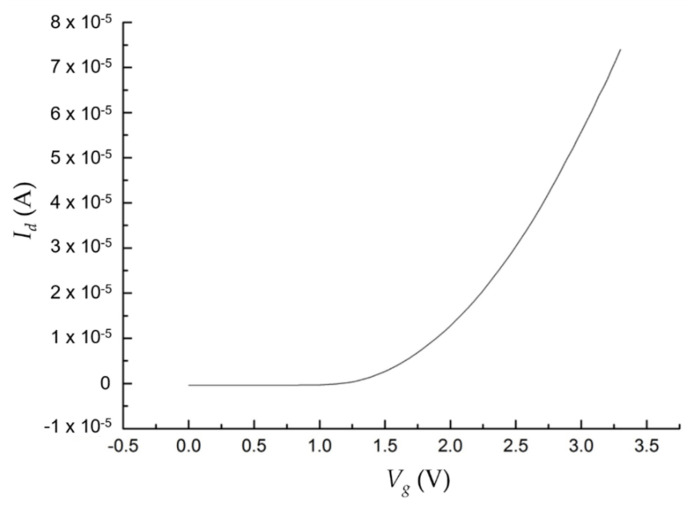
Measured dark turn-on characteristic (*I_d_*–*V_g_* curve) of the PDOSFET; *V_ds_* = 3.3 V.

**Figure 11:**
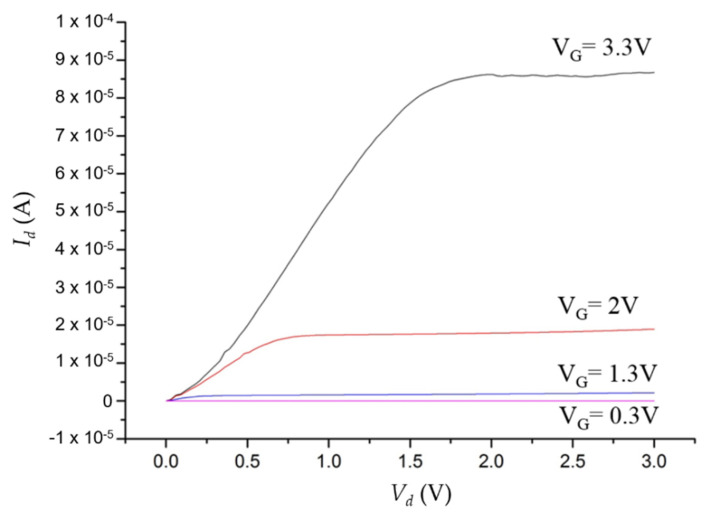
Measured dark *I_d_*–*V_d_* curve of the PDOSFET under various gate bias voltages.

The resultant PDOSFET looks just like a normal n-channel MOSFET (NMOSFET) except that a graphene layer is inserted between the n-type poly-Si gate and the gate oxide. That is, a PD is embedded right in the gate structure with minimal process modification. The device should be able to exhibit the normal dark electrical characteristics of an NMOSFET. To verify this, the *I_d_*-*V_g_* and *I_d_*-*V_d_* characteristics of the fabricated PDOSFET in the dark were measured by using an Agilent 4156a semiconductor parameter analyzer. Figure 10 shows the measured *I_d_*–*V_g_* curve of the PDOSFET under 3.3 V drain-to-source voltage (*V_ds_*). Normal turn-on behavior was observed. The threshold voltage for the channel to turn on is between 1 V and 1.5 V. By using the ATLAS device simulator from Silvaco, the threshold voltage of a MOSFET, the same as that for the PDOSFET shown in Figure 8 but without the embedded graphene layer, was calculated to be about 0.7 V. This MOSFET control device was fabricated and measured, and it showed a threshold voltage of about 0.85 V. The larger threshold voltage of the PDOSFET may be due to the existence of a depletion region in the poly-Si side of the graphene/poly-Si diode. It may absorb part of the applied gate voltage (*V_g_*) so that the control of the surface potential at the channel surface by V_g_ becomes less effective. Figure 11 shows the measured *I_d_*–*V_d_* characteristics of the PDOSFET under various gate bias voltages. Normal linear and saturation behaviors can be clearly recognized. The channel length of the fabricated PDOSFET is quite long; therefore, the short channel effect is hardly seen in Figure 11. From Figure 10 and Figure 11, it can be confirmed that the insertion of a graphene layer into the gate of a MOSFET does not destroy the properties of the transistor.

**Figure 12:**
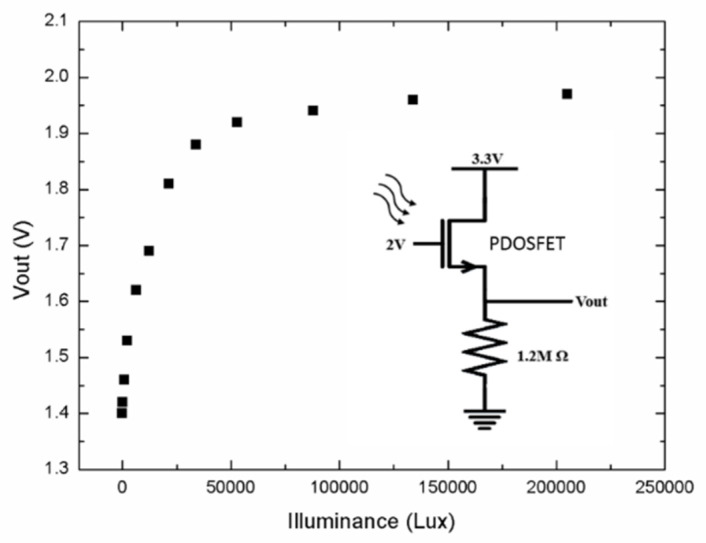
Measured PDOSFET source follower output voltage under various illuminance values.

When the PDOSFET is illuminated with light, the photovoltage developed at the PD should be able to effectively increase the gate voltage of the FET and thus increase the channel current. Therefore, when the PDOSFET is connected in a source follower configuration, the output voltage of the source follower should faithfully reflect the logarithmic behavior of the photovoltage of the PD. Figure 12 shows the measurement result for such a source follower when the PDOSFET was illuminated by a halogen lamp. The source follower connection is illustrated in the inset of Figure 12. It is clearly seen that the source follower output voltage (*V_out_*) shows a logarithmic dependence on illuminance. This indicates that both the graphene/n-type poly-Si junction PD and the FET integrated in the PDOSFET functioned well. Under low illuminance, V_out_ changes linearly with light intensity to provide good resolution. At high illuminance, *V_out_* is compressed to prevent saturation. The feasibility of using a single PDOSFET in a pixel to replace the P–N junction PD and the SF transistor is thus verified. It should be mentioned that the modification from the conventional 3T pixel structure to the new 3T pixel structure with the PDOSFET is very slight and that the responsivity of the graphene/poly-Si PD is mainly determined by poly-Si, not by graphene. Therefore, except for the optical input dynamic range, most other performance parameters such as the quantum efficiency, conversion gain, noise, and image lag of the new pixel should be similar to those of the conventional pixel.

Although the thickness of the poly-Si gate was designed to be 300 nm to absorb most of the visible light, it cannot be ruled out that some long wavelength light may still travel through the gate and reach the c-Si region under the gate oxide. These photons may still generate electron–hole pairs and contribute to the increase in the drain current. However, if this influence exists and dominates, the magnitude of the drain current increase should be linearly proportional to the increasing illuminance. Besides, the photo-generated drain current of the MOSFET control device (without PD at the gate) under 49,000 Lux illumination was measured to be only 0.1 μA even at *V_DS_* = 5 V. Therefore, the observed logarithmic dependence of *V_out_* (thus, the channel current) on illuminance is certainly not due to (at least not dominated by) the optical absorption in the c-Si substrate. Instead, the graphene/n-type poly-Si PD on top of the gate dominates the optoelectronic behavior of the PDOSFET source follower.

In addition, it was mentioned above that the source and drain areas of the PDOSFET were covered by metal in order to prevent the source and drain junctions from detecting light and contributing extra photocurrent. The effectiveness of this arrangement was investigated by measuring the dark and light drain currents when the gate voltage was set low to disable the channel. No change in the terminal current magnitude was observed with and without illumination. This confirms that the metal coverage on the source and drain regions effectively blocked the incident light.

**Figure 13:**
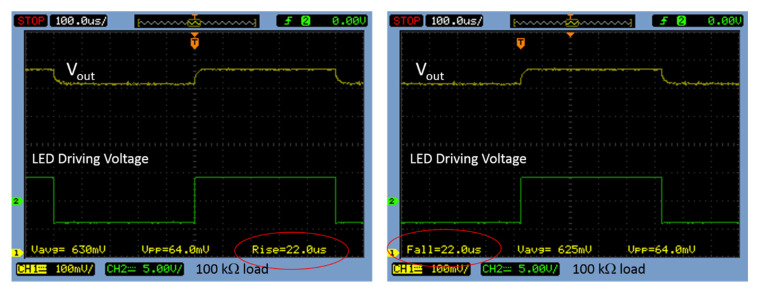
Measured transient behavior of the output voltage (*V_out_*) of the PDOSFET source follower with a loading resistance of 100 kΩ.

For most applications, it is desirable for the intrinsic operation speed of the phototransistor to be fast. Therefore, the transient response of the PDOSFET source follower was also investigated in this work. A white light-emitting diode (LED) was switched on/off by a voltage square wave to produce 5000 Lux light pulses onto the PDOSFET. The transient behavior of the source follower output voltage (*V_out_*) was observed. For the fabricated large, long-channel PDOSFET, both the rise time and the fall time of *V_out_* were as short as 22 μs when the loading resistance was 100 kΩ, as shown in Figure 13. When the loading resistance was increased to 1.2 MΩ, the rise time and the fall time only increased to 56 μs and 174 μs, respectively. This fast speed is beneficial to many applications.

One process issue about employing the proposed PDOSFET in mass production is the use of graphene in the front-end of standard, commercial CMOS processes. In such mass production processes, it is impossible to deposit the graphene layer by using slow techniques such as the transfer method. The direct wafer-level synthesis/deposition of graphene is needed. Besides, possible carbon contamination of the devices on the chip, due to the gases used in the graphene deposition process, must be avoided. Otherwise, graphene will not be usable in the front end of CMOS processes and it will be impossible to fabricate the PDOSFET. Although the new pixel structure shown in Figure 7 can still be implemented by using separate P–N junction diode and source follower MOSFET instead of using the PDOSFET, it is certain that the signal amplitude will be smaller and that the operation speed of the pixel will be slower because more parasitic capacitance emerges, and the conventional area competing problem between the PD and transistors will remain. Thus, the PDOSFET holds an important position in efficient HDR image detection. It is best for relevant front-end processes to be developed as soon as possible to facilitate the deployment of the PDOSFET.

**Figure 14:**
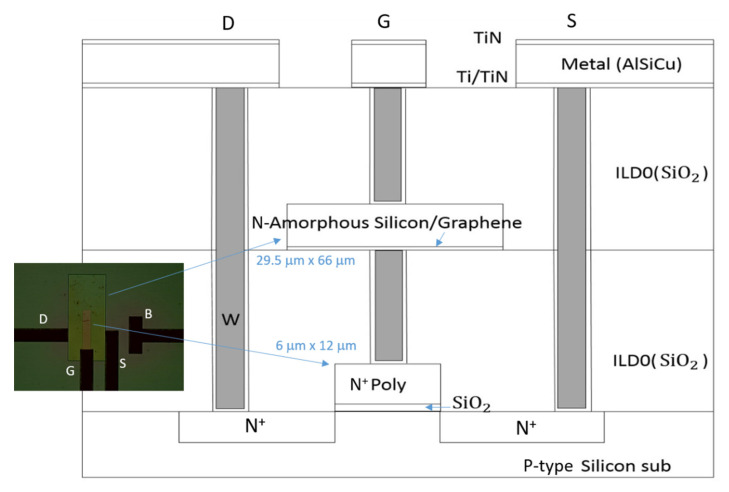
Schematic illustration of the Gr/a-Si PD and n-channel MOSFET (NMOSFET) connected structure. The PD is located above the NMOSFET, and the area of the PD is larger than that of the NMOSFET.

**Figure 15:**
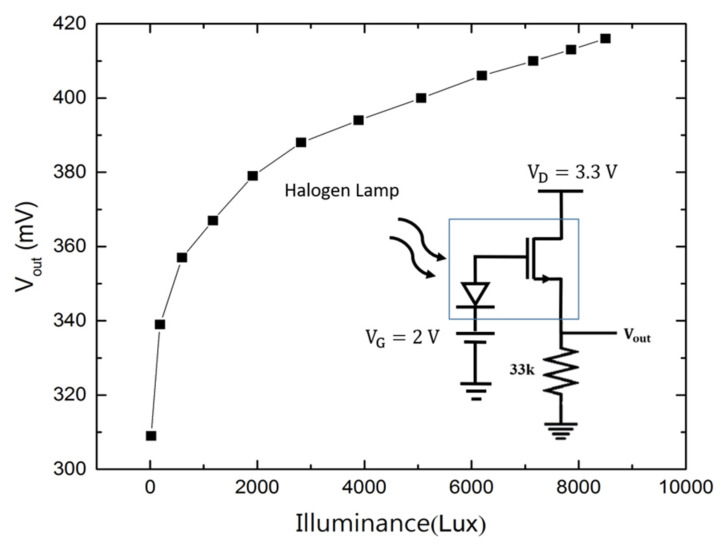
Measured Gr/a-Si PD and NMOSFET source follower output voltage under various illuminance.

As a remark, one possible way to implement the pixel structure shown in Figure 7 without using the PDOSFET to resolve the fill factor issue of the pixel is to use graphene/amorphous-silicon (Gr/a-Si) junctions as the PDs and fabricate these graphene/amorphous-silicon junctions during or after the back-end processes. In this way, although graphene is used, one can avoid producing contamination in front-end processes. The low-temperature process of depositing a-Si films also has minimal impact on the underlying CMOS devices. Since the graphene/amorphous-silicon PD and the transistors in the pixel do not locate in the same plane, they do not need to compete for chip area anymore. To demonstrate this alternative idea, we have designed and fabricated a Gr/n-type a-Si PD located above a Si NMOSFET, as shown in Figure 14. The graphene terminal of the Gr/n-type a-Si photodiode was connected to the poly-Si gate of the NMOSFET through a metal via. There is much more freedom for designing the area of the PD. In our samples, the a-Si was first deposited by using a hot wire chemical vapor deposition (HWCVD) system without doping. Its film thickness was about 150 nm. Then, phosphorus ion implantation was performed at 110 KeV of energy and a $1\times 10^{12}$ cm^−2^ dose to make it n-type. At the ohmic contact area, heavy doping was achieved by an additional ion implantation with 10 KeV of energy and a $2\times 10^{15}$ cm^−2^ dose. The area of the PD was designed to be $29.5\times 66{\;\mu m}^{2}$, much larger than the gate area ($6\times 12{\;\mu m}^{2}$) of the NMOSFET, to enhance the coupling of photovoltage to the gate of the NMOSFET. Figure 15 shows that the source follower circuit based on the sample in Figure 14 functioned very well. The logarithmic characteristic of the photovoltage was successfully revealed at the output node. Compared with the source follower circuit based on the PDOSFET as shown in Figure 12, the range of the output voltage span of this source follower circuit is smaller. This is not surprising, since metallic interconnect introduced more parasitic capacitance, which reduced the photovoltage magnitude. It may be possible to compensate this photovoltage reduction by enlarging the PD area so that its junction capacitance can dominate.

## 4. Conclusions

A graphene/n-type poly-Si junction PD was fabricated and characterized in this work. The dark *I*–*V* curve of the junction was found to be exponential, and the junction responded to light, with its photovoltage being a logarithmic function of illuminance. A new phototransistor called the PDOSFET, in which the graphene/n-type poly-Si junction PD was directly integrated with an n-channel MOSFET, was proposed, fabricated, and functionally verified. The channel current of the PDOSFET was efficiently modulated by the photovoltage of the PD and thus responded to light intensity logarithmically. The proposed PDOSFET shows great potential for CMOS image sensor applications, especially for detecting HDR images.
